# Visual dynamics: a WEB application for molecular dynamics simulation using GROMACS

**DOI:** 10.1186/s12859-023-05234-y

**Published:** 2023-03-22

**Authors:** Ivo Henrique Provensi Vieira, Eduardo Buganemi Botelho, Thales Junior de Souza Gomes, Roger Kist, Rafael Andrade Caceres, Fernando Berton Zanchi

**Affiliations:** 1 Laboratório de Bioinformática e Química Medicinal (LABIOQUIM), Fundação Oswaldo Cruz Rondônia, Porto Velho, RO Brasil; 2 Programa de Pós-Graduação em Biologia Experimental, Universidade Federal de Rondônia (UNIR), Porto Velho, RO Brasil; 3 FIOCRUZ Rondônia, Porto Velho, RO Brazil; 4 Universidade Federal de Ciências da Saúde de Porto Alegre – UFCSPA, Porto Alegre, RS Brasil; 5 Instituto Nacional de Epidemiologia na Amazônia Ocidental - EPIAMO, Porto Velho, RO Brasil; 6 Programa de Doutorado em Ciências – Cooperação IOC/Fiocruz Rondônia: Biologia Computacional e Sistemas (BCS), Porto Velho, RO Brasil

**Keywords:** Molecular dynamics, WEB, GUI, GROMACS, Python

## Abstract

**Background:**

The molecular dynamics is an approach to obtain kinetic and thermodynamic characteristics of biomolecular structures. The molecular dynamics simulation softwares are very useful, however, most of them are used in command line form and continue with the same common implementation difficulties that plague researchers who are not computer specialists.

**Results:**

Here, we have developed the VisualDynamics—a WEB tool developed to automate biological simulations performed in Gromacs using a graphical interface to make molecular dynamics simulation user-friendly task. In this new application the researcher can submit a simulation of the protein in the free form or complexed with a ligand. Can also download the graphics analysis and log files at the end of the simulation.

**Conclusions:**

VisualDynamics is a tool that will accelerate implementations and learning in the area of molecular dynamics simulation. Freely available at https://visualdynamics.fiocruz.br/login, is supported by all major web browsers. VisualDynamics was developed with Flask, which is a Python-based free and open-source framework for web development. The code is freely available for download at GitHub https://github.com/LABIOQUIM/visualdynamics.

## Background

The Molecular Dynamics (MD) is one of the techniques incorporated into bioinformatics, specifically by structural bioinformatics. With that, it is possible to obtain kinetic and thermodynamic characteristics of biomolecular structures. For example, macromolecular stability, identification of allosteric sites, elucidation of mechanisms of enzymatic activity, molecular recognition and properties of complexes with small molecules, association between proteins, protein folding and its hydration. Furthermore, MD enables a wide range of studies, including molecular design (widely used in drug design), in determining structure and its refinement (X-ray, NMR and protein modeling) [1]. The results obtained at the end of a MD are the richest and most complete in terms of non-quantum simulation [2]. However, implementing a MD experiment is not trivial [3].

Since its first applied approach to biology in 1977 much has evolved due to increased computational processing as well as improved coding. More than three decades ago, the first MD softwares that were intended for biological problems were launched: Gromacs [4], AMBER [5] and NAMD [6]. Since its first versions, these softwares still remain as the most used and cited. However, they continue with the same common implementation difficulties that plague researchers who are not computer specialists [3].

The paradigm of how software is executed has evolved as computing resources have improved. The first digital paradigm was the command line interfaces (CLI) followed by the evolution to the known graphical user interfaces (GUI) [7]. Following the evolutionary cycle, the interface produced by the World Wide Web (or simply WEB) is considered as an evolution of GUIs. These three paradigms currently co-exist depending on developers. Structural Bioinformatics software most commonly uses CLI and GUI paradigms. Some examples of classic software that use CLI are Modeller [8] for similarity modeling, Autodock [9] for molecular docking and Gromacs [4] for molecular dynamics. Examples of software that adopt the GUI type are SwissPDBviewer [10], Pymol [11], VMD [12], USCF Chimera [13], among others. With the emergence of HTML5 (Hypertext Markup Language version 5) [14], CSS (Cascading Style Sheets) [15], Javascript [16] technologies, among others, the development for WEB of many applications for structural bioinformatics could be rewritten to function in this new paradigm. Examples of similarity modeling web servers are: MODWEB [17] which uses Modeller as a back-end and Swissmodel [18]. Examples of web application servers for molecular docking are Haddock [19], Swissdock [20], Cluspro [21] and others.

While structural analysis, modeling and docking methodologies evolved from CLI paradigms to GUI and finally to WEB, MD continues mostly supported by command line execution (CLI type). Some good initiatives have emerged to improve this panorama. Examples of these initiatives are the implementation of plugins in existing software such as QwikMD [22] plugin to VMD, GROMACS Plugin to PyMOL [23] and the Molecular Dynamics Simulation option in UCSF Chimera [13]. Although the use of these plugins is an advance, their implementation is still a challenge for most unskilled researchers. It is common for difficulties to install and configure the adaptation that often compromise the full execution of the simulation.

Despite this, two excellent initiatives have emerged that facilitate the use of MD by beginning users of this technique: MDWEB [24], WebGRO [25]. MDWeb is a portal with several services for molecular dynamics. However, there is no possibility of performing molecular dynamics of protein–ligand complexes and the preparation of the dynamics has been a separate step of the execution. As mentinoed in their tutorial [24], it is necessary to download and modify the output script files from the preparation step. Just after these modificantions the MD can be performed. WebGRO, in turn, allows performing MD of protein–ligand complexes. However, using only the parameters assigned to the ligand by the PRODRG2 server [26], limiting the use of the GROMOS force field [27].

The objective of this work is to present a WEB tool developed to automate biological simulations performed in Gromacs. The application has two options of execution depending on the type of simulation you want to perform. The first is when the simulation is of an isolated biomolecule (Apoprotein) and the second when it is intended to simulate the dynamics of a macromolecule interacting with one small ligand (protein + ligand). Additionally, the user will be able to download the list of commands to run on their own server without any modification necessary. For researchers who want to implement it in their institutions, the application can be installed and served to other users via the WEB.

## Implementation

The application has characteristics of a classic WEB application in which a client terminal consumes the.html content in its station (front-end) provided by a server (back-end) through the internet (or a local network). The application was named VisualDynamics (VD) and uses the Queue-Request or Web-Queue-Worker architecture. The application is accessible through the electronic address http://visualdynamics.fiocruz.br/login and login access must be requested on the same website. Each user can only run one dynamic at a time. Just browse and enjoy the ease. VD offers you the possibility to leave your processes, disconnect from the server, and reconnect again later on, to check the progress of your projects. After completion of Molecular Dynamics, an email is sent to the user.If the goal is to implement a server, the components and libraries are all available for download from the project requirements list available on GitHub (https://github.com/LABIOQUIM/visualdynamics). In this case, the developer will need to install three main groups: (a) Gromacs 2018 (or higher) as shown in the developers’ portal (http://manual.gromacs.org/documentation/current/install-guide/index.html); (b) Python version 3.5 (https://www.python.org/), or higher and (c) The Flask micro framework and all libraries and dependencies (https://flask.palletsprojects.com/en/1.1.x/tutorial/layout/). Optionally, a virtual environment (virtualenv) was used to separate environments. All instructions and components for installing a new server are available on Github already mentioned. On the client side there are no special instructions.

## Results and discussion

**Fig. 1:**
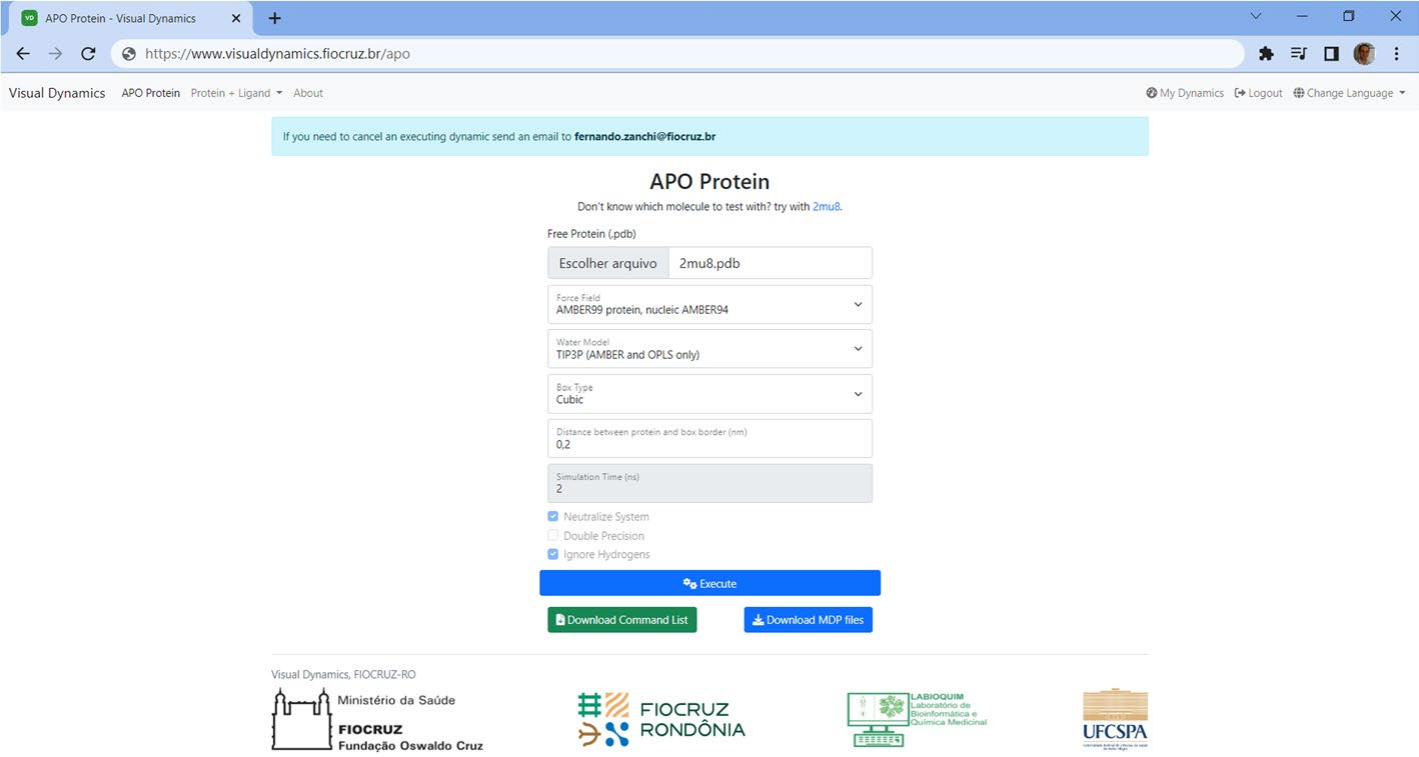
Example of a setup screen for MD of an apoprotein in VisualDynamics. Screenshot of the setup of molecular dynamics of an apoprotein contained in the file 2mu8 on VisualDynamics. The 2mu8.pdb file was uploaded and the parameters needed for execution were set. All files are available for download after the MD analysis step

**Fig. 2:**
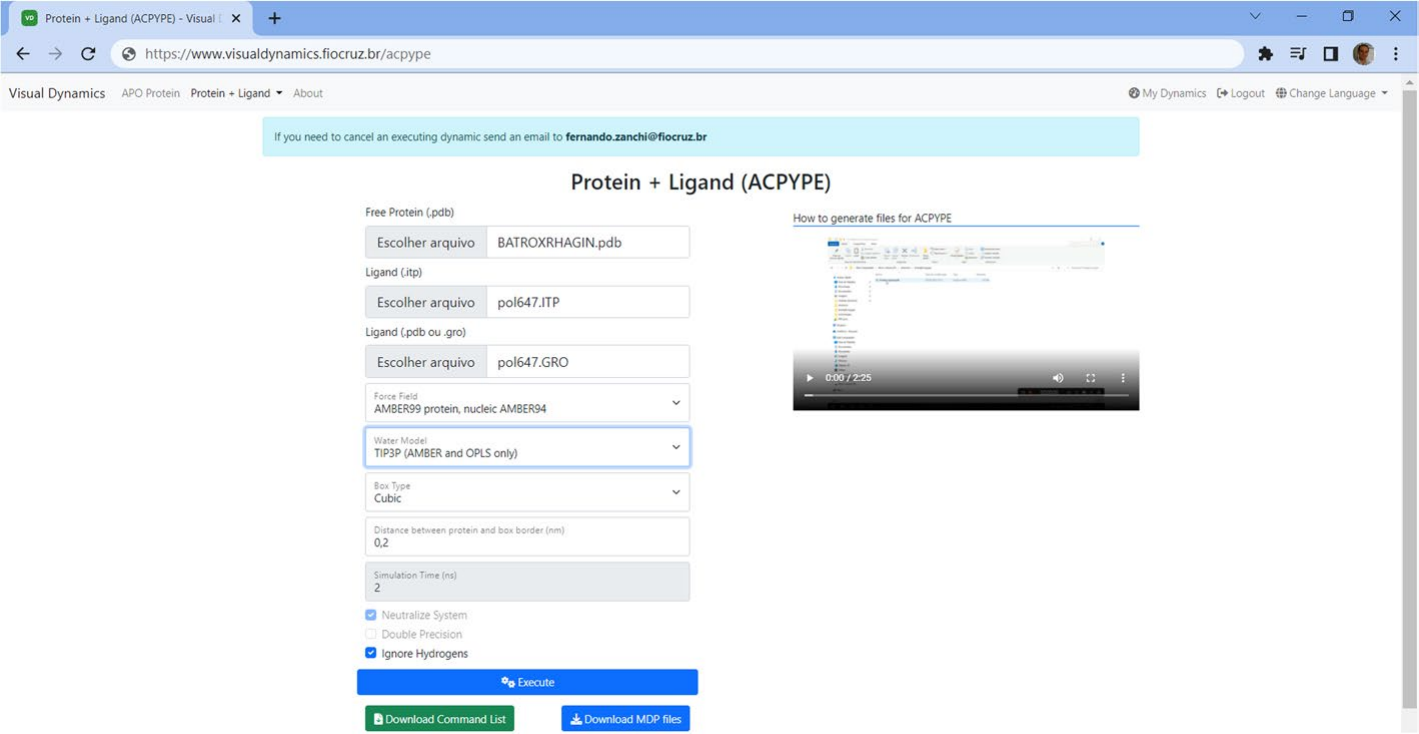
Example of a setup screen for MD of an protein–ligand conjugated in VisualDynamics. The VD screen shows the preparation of the molecular dynamics of the Batroxrhagin protein conjugated with the pol647 ligand. The BATROXRHAGIN.pdb, pol647.ITP, and pol647.GRO were uploaded and the parameters needed for execution were set. Next to it is a tutorial video explaining how to get the ligand files in ACPYPE. After MD analysis step, all files are available for download

The simulations follow all the classic steps of a preparation, solvation, neutralization, two-step minimization, two-step equilibration and finally production simulation [28]. The MD has been fixed at 2 ns, but can be changed in the software itself upon request by e-mail to the administrator before running. The application provides simulation in two options: isolated macromolecule (apoprotein) (Fig. 1) and conjugated macmacromolecule with a ligand (protein + ligand) (Fig. 2). Using an apoprotein option, we show the case study of a structure of twenty residues remaining belonging to the n-terminal region of protein-2 of the surface of the merozoite of *plasmodium falciparum* (PDB code 2mu8). The simulation on VD took about 32 min using the Amber99 force field with a cubic simulation box 2 Angstroms away from the protein boundary.

### Isolated macromolecule (apoprotein)

**Fig. 3:**
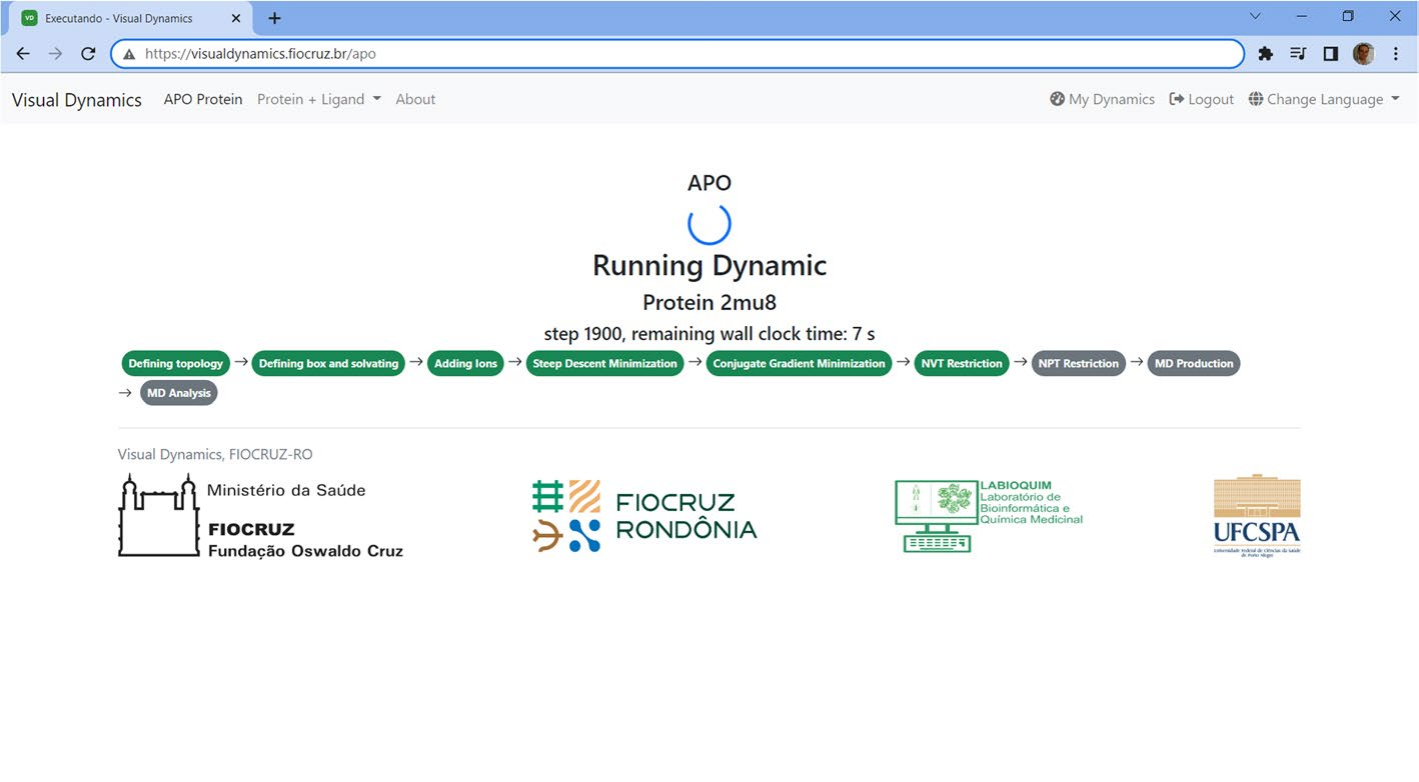
Screenshot of an apoprotein running in progress in VisualDynamics. Screen of executing molecular dynamics of apoprotein 2mu8.pdb on VisualDynamics. After MD analysis step, all files are available for download

Without any difficulty, the user uploads the.PDB file containing the biomolecule to be simulated, selects the force field, the water model, the type of simulation box, the distance of the biomolecule to the edge of the box, indicates if you want Gromacs to automatically neutralize the system and ignore the hydrogens from the original structure. Finally, alternatively, you have the option of downloading a text file containing all the commands in order of execution and analysis. This situation was considered when the user already has Gromacs installed in his laboratory and only wants to facilitate the execution of the simulation commands sequence. But if the researcher chooses to execute the complete dynamic on the web server, just click on 'Execute' and the server will execute it step by step (Fig. 3).

### *Conjugated macromolecule with a ligand (protein* + *ligand)*

There are two ways to generate the topology files (.itp file) and the structure's Cartesian coordinates file (.gro file) for simulation the biomolecule conjugated to a ligand: (a) with the ligand prepared in PRODRG2 server [26] or (b) ACPYPE server [29]. If the user has operational difficulty there are available some tutorial videos on the page. After obtaining the files, just upload them in the indicated fields, configure the desired parameters and run the simulation, or download the command list to run locally.

### Analysis and availability of results

**Fig. 4:**
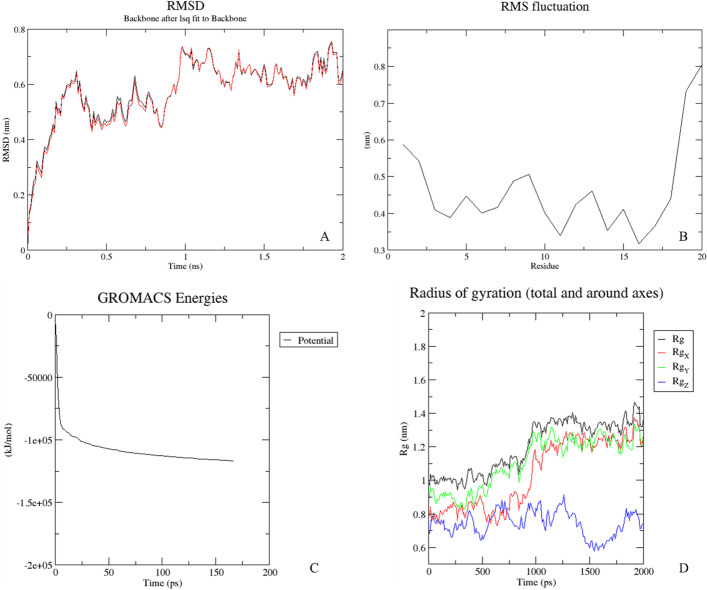
Examples of images generated in the analysis performed in VisualDynamics. The RMSD in **A**. RMSF is in **B**. The potential energy in the minimization step is in **C**. The radius of gyration of all axes and the average in **D**

After completing a dynamic, the trajectories are converted in order to centralize the biomolecule (gmx trjconv command). From these trajectories, many analyses are performed and compressed into a file that can be downloaded. This file contains the files in.png format generated by the Grace application [30] which reads the.xvg files produced according to each analysis application in the Gromacs package. This way, the user can quickly observe the results in the graphics in.png (Fig. 4). But in this case, the images come with low resolution.

Alternatively, the user can use the original files (.xvg) and submit them to another preferred software to generate images with better resolution (Table 1).

**Table 1:** Example of analysis input and output files

Input files	Gromacs package analysis	Output 1	Output 2
2mu8_sd_em.edr	Energy	2mu8_potentialsd.xvg	2mu8_potentialsd.png
2mu8_cg_em.edr	Energy	2mu8_potentialcg.xvg	2mu8_potentialcg.png
2mu8_nvt.edr	Energy	2mu8_temperature_nvt.xvg	2mu8_temperature_nvt.png
2mu8_npt.edr	Energy	2mu8_temperature_npt.xvg	2mu8_temperature_npt.png
2mu8_em.tpr and 2mu8.xtc	rms	2mu8_rmsd_cris.xvg	2mu8_rmsd_cris.png
2mu8_pr.tpr and 2mu8.xtc	rms	2mu8_rmsd_prod.xvg	2mu8_rmsd_prod.png
2mu8_pr.tpr and 2mu8.xtc	gyrate	2mu8_gyrate.xvg	2mu8_gyrate.png
2mu8_pr.tpr and 2mu8.xtc	rmsf	2mu8_rmsf_residue.xvg	2mu8_rmsf_residue.png
2mu8_pr.tpr and 2mu8.xtc	sasa	2mu8_sas_residue.xvg	2mu8_sas_residue.png

Input files are generated after executing an ‘mdrun’ command and depend on each specific step. Output 1 and 2 files will be made available in the zipped file for download. Output 2 refers to files generated after running application ‘grace’ or ‘xmgrace’ over.xvg files as instructed in the Gromacs manual. In this example, the input and output files refer to a protein contained in the 2mu8.pdb file uploaded in the initial screen.

The researches can also download the Run Log files online. Furthermore, can download all.mdps files used in the simulation online. As previously mentioned, the VD allows the user to choose to generate a command script file for download instead of running it on our servers locally. To do this just click on “Download command list”. This option is useful when the user already has Gromacs installed in his computer park and only wants to speed up, in a standardized way, the simulation process of a biomolecule and/or a biomolecule conjugated with a ligand. This file contains the list of ordered commands and all parameters filled out. Simply batch run it on Linux or copy and paste each line and run at the prompt.

## Conclusions

The learning curve for implementing a MD is long. There is a lot of knowledge added in the technique. Even for computationally experienced users, the challenge persists and advances into other areas that are often limiting the success of the simulation. Aspects brought by molecular biophysics, chemistry or physical chemistry make up many obstacles to be overcome. VisualDynamics brings agility in the execution of in silico MD experiment using Gromacs. Inexperienced users with no computer skills will be able to produce dynamics and manage of the data produced at the end of the dynamics. Whether in online or scripted form, the application advances towards allowing the community of researchers interested in applying the MD in their analysis without worrying about intermediate details.

In addition, we are continuously working so that future versions bring more options for parameterization of no-protein ligands using other force field options and other topologies with more improved loading methods. The group's objective is also to implement its own methodology to calculate partial charges and topologies directly on the software, thus allowing to reduce steps.

## Availability and requirements

Project name: VisualDynamics. Project home page: http://visualdynamics.fiocruz.br/. Operating system: Linux. Programming language: Python 3.5 or higher, Flask, HTML5. Other requirements: GROMACS 2018 or higher; Grace or Xmgrace. License: FreeBSD. Any restrictions to use by non-academics: None.

## Data Availability

All sources codes are freely available at https://github.com/LABIOQUIM/visualdynamics.

## Abbreviations

MD: Molecular dynamics
VD: VisualDynamics
CLI: Command line interfaces
GUI: Graphical user interfaces
WEB: World Wide Web
VMD: Visual molecular dynamics
ACPYPE: Antechamber python parser interface
